# Multimodal Approaches Based on Microbial Data for Accurate Postmortem Interval Estimation

**DOI:** 10.3390/microorganisms12112193

**Published:** 2024-10-30

**Authors:** Sheng Hu, Xiangyan Zhang, Fan Yang, Hao Nie, Xilong Lu, Yadong Guo, Xingchun Zhao

**Affiliations:** 1 Institute of Forensic Science, Ministry of Public Security, Beijing 100038, China; hsheng2018@163.com (S.H.); yangfan6616@126.com (F.Y.); oliverepoch@aliyun.com (H.N.); 18638129955@163.com (X.L.); 2 Department of Forensic Science, School of Basic Medical Sciences, Central South University, Changsha 410013, China; zxy196@csu.edu.cn (X.Z.); gdy82@126.com (Y.G.)

**Keywords:** rupture, postmortem interval, microbiome, multimodal

## Abstract

Accurate postmortem interval (PMI) estimation is critical for forensic investigations, aiding case classification and providing vital trial evidence. Early postmortem signs, such as body temperature and rigor mortis, are reliable for estimating PMI shortly after death. However, these indicators become less useful as decomposition progresses, making late-stage PMI estimation a significant challenge. Decomposition involves predictable microbial activity, which may serve as an objective criterion for PMI estimation. During decomposition, anaerobic microbes metabolize body tissues, producing gases and organic acids, leading to significant changes in skin and soil microbial communities. These shifts, especially the transition from anaerobic to aerobic microbiomes, can objectively segment decomposition into pre- and post-rupture stages according to rupture point. Microbial communities change markedly after death, with anaerobic bacteria dominating early stages and aerobic bacteria prevalent post-rupture. Different organs exhibit distinct microbial successions, providing valuable PMI insights. Alongside microbial changes, metabolic and volatile organic compound (VOC) profiles also shift, reflecting the body’s biochemical environment. Due to insufficient information, unimodal models could not comprehensively reflect the PMI, so a muti-modal model should be used to estimate the PMI. Machine learning (ML) offers promising methods for integrating these multimodal data sources, enabling more accurate PMI predictions. Despite challenges such as data quality and ethical considerations, developing human-specific multimodal databases and exploring microbial–insect interactions can significantly enhance PMI estimation accuracy, advancing forensic science.

## 1. Introduction

Accurately determining the postmortem interval (PMI) is crucial in forensic investigations of unnatural deaths, as it helps establish the nature of the case, provides investigative leads, and offers critical evidence for trials [1]. Early postmortem changes such as body temperature, rigor mortis, livor mortis, and algor mortis can effectively estimate the early PMI [2]. However, in the later stages of decomposition, these external signs become difficult to quantify, making late-stage PMI estimation a significant forensic challenge.

Decomposition is typically divided into five stages: fresh, bloat, active decay, advanced decay, and skeletonized stage [3]. While these stages summarize physicochemical changes, they are subjective and do not represent discrete sequences. Therefore, finding objective, accurate, and reproducible indicators for stage division is essential for improving late-stage PMI estimation. Microorganisms play a dominant role in decomposition [4]. After death, the body’s immune system ceases to function, allowing microbes to proliferate rapidly [5,6]. The microbial communities driving decomposition are somewhat predictable across different hosts and environments, potentially enabling the use of microbial sequences to estimate PMI [7].

Anaerobic microorganisms transform carbohydrates, lipids, and proteins into organic acids (e.g., propionic acid, lactic acid) and gases (e.g., methane, hydrogen sulfide) [8]. The internal pressure from gas accumulation forces purge fluids to escape from cadaveric orifices and seep into the soil [9]. This leads to changes in the microbial communities of the skin and soil. These changes, including a localized flush of microbial biomass, shifts in soil fauna, carbon mineralization, and increased soil nutrient status, could serve as objective criteria for marking the onset of the rupture stage within the bloat stage [10]. Additionally, the transition of the internal organ microbiome, especially the gut, from anaerobic to aerobic communities could signify the end of the rupture stage [10]. Thus, we propose a concept of rupture point different from the appearance of visible cracks in the cadaver, which is that communication between the internal and external environment of the cadaver can be realized by changing the tissue permeability. At this time, the macro signs of the cadaver can only reveal the gradual collapse of the abdomen of the cadaver, while microscopic changes in the abundance of microorganisms can be observed. This allows decomposition to be divided into two objective stages: pre- and post-rupture [11].

**Figure 1:**
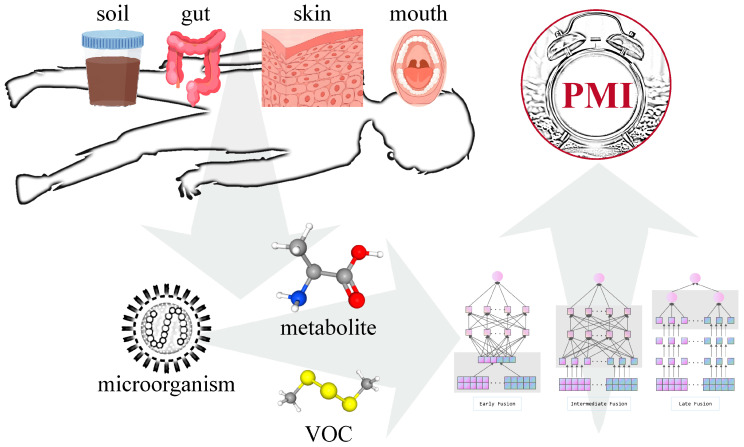
PMI estimation process based on relevant microbial markers.

Previous research has mentioned the shift from anaerobic to aerobic bacteria as a key transition but has not leveraged this in PMI estimation models [12]. Current single regression models using microbial evidence fail to capture the complex, nonlinear relationships within the decomposition process, treating it as a single linear progression. This limitation hinders the accuracy of PMI estimation using microbial data. While both theory and case studies support the feasibility of using microorganisms for PMI estimation, inflexion points in microbial community changes during decomposition make the current unsegmented models inadequate. Additionally, single-modal microbial evidence is insufficient. Alongside microbial shifts, the changes in nutrients, small molecule metabolites, volatile gases, and physicochemical properties within the body and environment provide a comprehensive view of the decomposition process. Integrating these changes can enhance PMI estimation accuracy, offering a richer information base for forensic investigations (Figure 1).

## 2. Microbiology Changes During Decomposition

After death, the human microbiome undergoes significant changes over time, especially during the rupture stage when major shifts in microbial communities occur [13,14,15]. Microorganisms interact with the corpse at different stages in distinct ways [16]. During the bloat stage, endogenous anaerobic bacteria in the gut, such as Lactobacillaceae from the Firmicutes phylum and Bacteroidaceae from the Bacteroidetes phylum, migrate into the abdominal cavity and proliferate extensively [17]. They produce gases containing hydrocarbons, organic amines, and ammonia, which primarily contribute to the bloating of the body [17]. Further decomposition and tissue dissolution are driven by gut-derived microbes and those spread via blood and lymphatic fluids. As the body ruptures, aerobic bacteria and facultative anaerobes, such as Flavobacterium, Pseudomonas, Brucella, and Enterobacteriaceae, become the dominant decomposers [18]. Eventually, environmental fungi, including Mucor, Geotrichum, and Penicillium, colonize the remaining body tissues, forming mold spots and mummification [19]. Different organs or body parts host distinct microbial communities, but the succession of these communities changes markedly after death and is closely linked to the time since death. In general, bodies with shorter postmortem intervals are dominated by facultative anaerobes, while those with longer intervals are mainly decomposed by obligate anaerobes [20,21]. During the skeletonization stage, spore-forming microorganisms become predominant. Overall, the progression of microbial succession provides valuable insights into the postmortem interval, with different stages of decomposition marked by shifts in microbial communities.

The microbiome of a body undergoes significant changes before and after rupture [10,15,21,22,23,24]. Hyde et al. proposed that this transition marks the shift from early to late decomposition. This “purge” event is associated with opening the abdominal cavity to the environment, during which larvae clear away much of the tissue, potentially accelerating the decay process. Zhao et al. introduced the concept of the “rupture point,” noting that there are significant differences in the signs of decomposition, microbial community structure, and physicochemical properties of the environment before and after the natural rupture of the abdominal cavity [22]. These differences are highly observable and can be accurately assessed through forensic, biological, and physicochemical methods. Building on previous research, a refined concept of the “rupture point” is proposed: the moment when the internal environment of the body starts exchanging with the external environment. This exchange can occur through changes in tissue permeability rather than visible cracks. Macroscopically, this may be observed as the gradual collapse of the abdominal area, while microscopically, changes in microbial abundance can be detected. Thus, the rupture point offers a precise marker for segmenting the stages of decomposition.

Previous research indicates a significant shift in the microbial communities of the gut and surrounding soil at a distinct time point during decomposition, around 24 to 27 days postmortem [22]. For winter soil samples, the Shannon index shows no difference in the first 24 days but fluctuates after this period. The Shannon index remains stable for rectal samples during the initial 24 days and then gradually decreases. In gut samples, the relative abundance of Acidobacteria, Actinobacteria, Chloroflexi, and Bacteroidetes decreases after 24 days, while the relative abundance of Proteobacteria increases. Soil samples show a decrease in Vicinamibacteraceae_norank and Lactobacillus after 24 days, with an increase in Lactobacillus from 24 to 36 days. Hyde et al. found a significant shift in microbial communities on different body parts around the third day postmortem, marking the transition from early to late decomposition, often accompanied by abdominal bloating and rupture [24,25]. Before this point, the skin microbiome on the cheeks, biceps, and torso is dominated by Proteobacteria, which later shifts to Firmicutes and Actinobacteria. The oral microbiome shows a similar trend. However, the gut microbiome shifts from being dominated by Firmicutes and Bacteroidetes to Proteobacteria. Overall, as decomposition progresses, microbial diversity decreases, and abundance increases. Metcalf et al. confirmed the high correlation between microbial succession and postmortem interval (PMI) in mouse models, with microbial diversity negatively correlated with PMI and abundance positively correlated [20,21,26].

The gut microbiome is a primary source of postmortem microbial activity, often used in research due to its stability [9]. Clostridium species in the gut are key markers postmortem and are part of the “postmortem Clostridium effect” (PCE), which is significant for PMI estimation [27]. Researchers consistently find that Firmicutes, Bacteroidetes, and Proteobacteria dominate decomposition. As decomposition progresses, the relative abundance of Firmicutes decreases while Bacteroidetes and Proteobacteria increase. Zhao et al. identified day 24 postmortem as a turning point, with Bacteroidetes declining and Proteobacteria rising [22]. Additionally, Acidobacteria, Actinobacteria, and Chloroflexi show a decreasing trend in relative abundance after day 24 [22]. Hyde found that the abundance of Bacteroidetes and Parabacteroides in human proximal colon samples negatively correlates with PMI [9]. Guo observed a decrease in Firmicutes and Bacteroidetes, with an increase in Proteobacteria during decomposition [28]. Another study noted that Firmicutes had the highest average relative abundance at 0 days (nearly 80%) and consistently remained the most abundant across all time points despite a general decline. Bacteroidetes increased until day 9, then decreased, while Proteobacteria peaked at 12, 18, and 21 days postmortem [29].

During the early stages of decomposition, Bacillus, Lactobacillus, and Streptococcus dominate the microbial community. As decomposition progresses, anaerobic bacteria such as Clostridium, Proteobacteria, and Ignatzschineria become more prevalent [20,27,30,31]. Zhao et al. identified a critical transition point in the microbial community composition. Initially, the gut microbiota is primarily composed of Muribaculaceae_norank, Lactobacillus, Limosilactobacillus, and Yersinia [22]. Muribaculaceae_norank declines after 18 days, while Lactobacillus peaks between 24 to 33 days. Yersinia becomes dominant by day 27, surpassing Lactobacillus by day 30.

In later stages, Proteobacteria and Ignatzschineria from the Gammaproteobacteria class dominate the oral and rectal microbiota [28]. Metcalf et al. noted that Enterobacteriaceae, often associated with wastewater and animal matter, becomes more abundant after tissue rupture [10]. Proteobacteria are commonly linked to meat decomposition and have been found on animal hides in slaughterhouses [32]. Bacteroidetes and Firmicutes are reported as the two primary phyla in the human gut (intestines, rectum, and cecum) and fecal samples. Actinobacteria, widely distributed in terrestrial and aquatic ecosystems, play a crucial role in decomposing recalcitrant biological materials [33].

Research has also explored the relationship between fungal communities and cadaver decomposition [34,35,36], which, although less studied, is significant in advanced decomposition stages such as skeletonization. Metcalf et al. demonstrated that PMI estimation models based solely on fungal communities are as effective as those based on bacterial communities [10]. Fu et al. studied fungal composition and succession in decomposing pig carcasses indoors and outdoors, finding that Candida xylopsoci, Ascomycota sp., and Thermoascus aurantiacus dominate the fungal community in decaying corpses [37]. These specific fungal taxa can be linked to PMI estimation as reliable indicators.

In summary, the decomposition process involves a dynamic succession of microbial communities. Early stages feature aerobic and facultative anaerobes, while strict anaerobes dominate later stages. This microbial succession provides critical PMI estimation data, with bacterial and fungal communities offering valuable insights.

## 3. Metabolic Changes During Decomposition

When a body is subjected to physiological, pathological, or pharmacological stimuli, its metabolic network experiences spatial and temporal changes [38]. Metabolomics aims to study these variations to reverse-engineer the influencing factors and their potential impacts on the body [39,40]. After death, metabolic changes continue until the complete cessation of all biochemical activities. Metabolomics is well-suited for studying time-related and diagnostic phenomena with its non-destructive, holistic, and dynamic nature [40].

In the early postmortem period, transcriptional regulation can still influence metabolic changes [41]. For instance, in heart tissue, levels of phenylalanine and some branched-chain amino acids increase, while niacinamide, IMP, ATP, and inosine decrease. In kidneys, phenylalanine and tyrosine levels significantly rise within the first 24 h. Skin tissue shows increased lactate levels six hours after death, likely due to bacterial activity, and decreased glucose and taurine levels [42]. Early metabolic changes are primarily associated with energy metabolism and DNA synthesis, whereas later changes relate more to microbial metabolism [15].

As decomposition progresses, various metabolites are consumed, produced, and accumulated in tissues [43,44]. In brain tissue, researchers have used animal models to identify metabolites that can help estimate PMI. In 2005, Scheurer et al. used NMR analysis on sheep brains at a constant temperature for 18 days, identifying five metabolites (acetate, alanine, butyrate, trimethylamine, and propionate) that correlated well with PMI within 250 h [45]. Further studies in 2011 expanded this to eight metabolites (including aspartate, GABA, and valine) across different temperatures, showing a high correlation between estimated and actual PMI [46].

Commonly detected amino acids in soils associated with decomposition include creatine, alanine, proline, taurine, and GABA [47]. Creatine levels notably increase in blood and tissues during the early postmortem interval, while taurine is abundant in bile and intestines. Alanine and proline, common in vertebrate proteins, have been detected in the early stages of decomposition. Human cell autolysis produces amino acids and ammonium early in decomposition, but elevated microbial respiration and protease activities indicate significant microbially mediated decomposition. Frequent soil metabolites include anthranilate, creatine, 5-hydroxyindoleacetic acid, taurine, xanthine, N-acetylglutamine, acetyllysine, and sedoheptulose 1/7-phosphate [47]. Lipids such as phosphatidylethanolamine and monogalactosyldiacylglycerol are also prevalent. Decomposition soils are enriched in metabolites related to amino acid metabolism and the TCA cycle [47]. Compared to pig soils, elevated metabolites in human decomposition soils include 2-oxo-4-methylthiobutanoate, sn-glycerol 3-phosphate, and tryptophan. Lactate is a notable metabolite modulated in various matrices. Under anaerobic conditions, lactate accumulates due to glycolysis [42]. For example, O. alkaliphila can cross-feed with Ignatzschineria, Acinetobacter, Savagea, and Vagococcus lutrae, donating amino acids like aspartate, isoleucine, leucine, tryptophan, and valine, along with sn-Glycero-3-phosphoethanolamine.

## 4. Volatile Organic Compounds (VOCs) Changes During Decomposition

Influenced by enzymes, microorganisms, and scavenging animals, the decomposition process releases various volatile organic compounds (VOCs) [48,49]. These VOCs are byproducts or end products of decomposition activities, contributing to the characteristic “death odor” associated with decaying bodies [50].

In 2004, Vass et al. conducted the first study on VOCs released during decomposition [50]. The study aimed to identify the odor characteristics of fresh and aged bodies, establishing a “decomposition odor analysis database” with over 400 identified VOCs across eight categories. This pioneering work laid the foundation for subsequent research into human decomposition odors. In 2005, Statheropoulos et al. analyzed VOCs from highly decomposed bodies retrieved from the sea (estimated PMI of 3–4 weeks) [51]. Using sorbent tubes (ST) to collect VOCs, which were then analyzed via gas chromatography-mass spectrometry (GC-MS), the study identified and quantified over 80 VOCs, with primary components including dimethyl disulfide (DMDS), toluene, n-hexane, 1,2,4-trimethylbenzene, acetone, and 3-pentanone.

In a subsequent study, Statheropoulos et al. analyzed VOCs during the early stages of decomposition, using similar sampling and analysis techniques [51]. They detected over 30 VOCs in early decomposition, with 11 core substances (ethanol, acetone, DMDS, toluene, octane, 2-butanone, methyl ethyl disulfide, dimethyl trisulfide (DMTS), and ortho-, meta-, and para-xylene) consistently detected within the first 24 h. The findings indicated a significant increase in the total number of detectable VOCs over time: approximately 30 VOCs in bodies with a PMI of 3 days, and around 80 VOCs in bodies with a PMI of 3–4 weeks.

Certain VOCs, such as DMDS and trimethyl disulfide, are associated with bacterial metabolism and can attract or repel insects [52,53]. DMDS is emitted by various microorganisms, including Pseudomonas [54] and Proteus [55], and can attract Chrysomya rufifacies [53]. DMTS is emitted by Streptomyces [56], and Cochliomyia macellaria is attracted to DMTS on cadavers [57]. Sulfur-containing VOCs, major contributors to decomposition odors, are present from the bloating stage into active decay. Bacteria emit different VOCs during decomposition, such as indole, significantly correlated with families Planococcaceae and Tissierellaceae [58].

The number of VOCs likely peaks during bloating due to heightened microbial activity and potential gas leakage before the rupture event. While sulfur-containing VOCs are present throughout all decomposition stages [59], aldehydes are more commonly observed post-rupture [60,61,62]. This indicates a dynamic change in the VOC profile as decomposition progresses, with microbial activity driving the production and release of various VOCs.

The study of VOCs during decomposition provides valuable insights into the chemical processes underlying the decomposition process and offers the potential for improving postmortem interval (PMI) estimation. By understanding the specific VOCs associated with different stages of decomposition, forensic scientists can develop more accurate and reliable methods for determining the time since death.

## 5. Multimodal Analysis

In the early stages of decomposition, endogenous gut bacteria dominate the breakdown process by digesting macromolecules. As the body ruptures, microbes from the skin and soil thrive on the abundant nutrients, leading to explosive growth. This microbial activity, coupled with the chemical breakdown of proteins, sugars, and fats within cells, results in pH changes in the decomposition fluids, further influencing the composition of microbial communities. This process is marked by significant changes in nutrient levels and oxygen content within the body [63]. As previously mentioned, microorganisms and their metabolic products, including VOCs, exhibit distinct expression patterns before and after the rupture, significantly altering their profiles [15,52,53]. However, current studies often consider only single-dimensional variables, failing to comprehensively reflect real-case scenarios.

The necrobiome, which includes biological and ecological processes governed by bacteria [64], insects, and vertebrates [65], is a fundamental theoretical basis for integrating decomposition theory with PMI determination [66]. This broad framework also considers interactions with abiotic factors, soil, and the surrounding environment. Collaborating across multiple disciplines can advance our understanding of decomposition science and develop a more comprehensive and holistic perspective of this crucial ecological process [67,68]. Interdisciplinary collaboration between microbial ecology, chemistry, and forensic science experts can explore the synergistic effects, feedback loops, and complex interactions driving decay variation. Forensic science is crucial for justice administration, and researchers advocate for systematic, reliable, and affordable collaborative research. Despite the availability of data on microbiology, small molecule metabolites, and VOCs (Table 1), they are rarely integrated, and few advances have been reported in computationally exploiting the research potential of large-scale, multimodal integration. Some studies have attempted multi-omics integration strategies for PMI estimation, using different markers (e.g., proteins, metabolites, and lipids) to assess both short- and long-term PMIs with high accuracy. These markers have complementary decay rates, offering the advantage of covering both short-term PMIs (with metabolites and lipids) and long-term PMIs (with more stable proteins).

Machine learning (ML) techniques have enormous potential to convert data into diagnostic and prognostic models [72]. Multimodal data fusion strategies include early fusion, intermediate fusion, and late fusion [72,73,74,75]. Early fusion integrates input data at the input layer, with no differentiation of feature origins. This approach is simple but may fail to identify higher-level relationships between modalities, and it is sensitive to different sampling rates. Intermediate fusion learns and fuses feature vectors through neural networks. This strategy offers flexibility in finding the right depth and sequence for fusing features. Late fusion combines decisions from separate unimodal submodels into a final decision. It is suitable for heterogeneous modalities and is not affected by input dimension imbalances, but it cannot learn interactions between features of different modalities.

The medical setting is often limited by the disparity between data availability and the data needed to fit multimodal models [76]. Methodological advancements focus on increasing robustness to overfitting and dealing with missing data rationally. A significant design choice for multimodal approaches is the extent to which each data input should be modeled before encoding joint representations. Although no literature has been found using multimodal ML to estimate PMI, multimodal patient stratification using complementary multi-omics cancer data is well-developed [77,78,79]. For example, integrating bulk transcriptomics, microRNA (miRNA) sequencing, and promoter methylation status with early fusion autoencoders has enhanced the ability to stratify patients with hepatocellular carcinoma [77]. Several multi-omic models also incorporate traditional clinical features, such as age and hormone receptor status, to stratify patients with breast cancer by overall survival more accurately than unimodal models [78].

By inputting multimodal data and using artificial intelligence for modal integration, the model can predict both pre- and post-rupture stages and the PMI, which involves a multimodal multitask model. The model can classify the death time as occurring before or after rupture, optimizing the PMI estimation process. Previous studies have used multitask theory (piecewise) for PMI prediction, combining classification and regression models. For instance, a two-layer model based on bacterial succession achieved 90.48% accuracy in distinguishing PMI groups (0–7 days and 9–30 days) and yielded a mean absolute error of 0.580 days within 7 days and 3.165 days within 9–30 days [80]. Future studies should focus on further developing and validating these multimodal ML approaches for PMI estimation.

## 6. Future Outlook

Research on estimating postmortem intervals (PMIs) based on microbial evidence and associated metabolic changes is still in its infancy and cannot be directly applied to forensic practice. If significant multimodal data changes (microbial, metabolic, and volatile organic compounds) can be linked to the objective phenomenon of body rupture, it could provide reliable anchors for accurately estimating PMI by dividing the later stages of death into pre- and post-rupture periods, just like the rupture point we proposed which could divide the stages of decomposition objectively. Machine learning (ML) offers effective methods for integrating multimodal data features and can support multitask models that simultaneously address classification (pre-rupture vs. post-rupture) and regression (PMI estimation), simplifying the complexity of using rupture stages to infer PMI. However, several issues warrant further discussion.

### 6.1. Construction of Human Multimodal Databases

Current research focuses mainly on non-human animals such as pigs, rats, mice, and rabbits. The dominant microbial species or metabolic products in these animals differ from those in human bodies. It is essential to collect multimodal data (microbial, metabolic, and volatile organic compounds) from human corpses, adhering to ethical standards. Further understanding the interconnections within multimodal data, exploring interactions between microbes, and examining the transformations between microbial and corpse-derived metabolites will provide more resounding theoretical support for constructing comprehensive multimodal databases.

### 6.2. Challenges and Opportunities of ML

Despite its potential, ML in forensic microbiology faces significant challenges. Obtaining high-quality training data is difficult; data imperfections require substantial cleaning and normalization. High-dimensional datasets with few instances and unbalanced target attributes complicate model generalization. Techniques like regularized models, feature extraction, and fine-tuning pre-trained deep neural networks can mitigate these issues. Multidisciplinary research necessitates long-term collaboration for effective communication and data-sharing. ML algorithms will integrate into workflows like Galaxy and Bioconductor as they mature, enhancing accessibility. Advanced ML, particularly deep learning, can manage large datasets of images, text, and genomic sequences, offering opportunities to solve previously intractable problems. Microbiologists must either embrace ML or leave it to specialists.

### 6.3. Interaction Between Microbial and Insect Evidence

Microbes and necrophagous insects are key biological factors driving body decomposition. Existing research often overlooks the interactions between microbes and insects. When both are present, the traditional microbial succession patterns and insect development stages used to infer PMI may lead to larger errors, violating the precision required in forensic science. Considering the interactions among insects, microbes, and corpses comprehensively and reconstructing insect–microbial evidence databases are essential for developing a high-precision PMI estimation system in forensic science.

## Figures and Tables

**Table 1:** Research based on relevant microbial markers for predicting PMI.

Marker	PMI	Experimental Model	Analysis Model	Model Performance	References
Microbiology					
	48 d	mice skin	RF	MAE 3.30 ± 2.52 d	[10]
	5 d	pig skin/mouth	RF	94.4% accuracy rate	[26]
	579 d	porcine rib	RF	RMSE ± 104 d	[69]
	40 d	pigs gravesoil/rectum	RF	MAE 1.375 to 2.478 d	[11]
	21 d	human face	RF	residual 26.47ADD	[70]
	21 d	human hip	RF	residual 28.59ADD	[70]
	71 d	mice gravesoil/cecum/skin	RF	RMSE 342.14ADD	[15]
Non-volatile metabolites					
	144 h	human liver	SNN	accuracy = 92.4%	[71]
	420 h	sheep brain	linear\quadratic\three-parameter-logistic functions	- *	[45]
	5–21 w	humans and pigs gravesoil	PLS-DA	-	[47]
Volatile organic compounds					
	5 d	pig	-	-	[59]
	43 d	pig	-	-	[60]
	56 d	pig	-	-	[62]

* “-” means not mentioned in the reference.

## Data Availability

Not applicable.
